# Supplementary figures and images for: Genetics of white color and iridophoroma in “Lemon Frost” leopard geckos
Source: PLoS Genet. 2021 Jun 24;17(6):e1009580. doi: 10.1371/journal.pgen.1009580 (PMC8224956; doi:10.1371/journal.pgen.1009580)

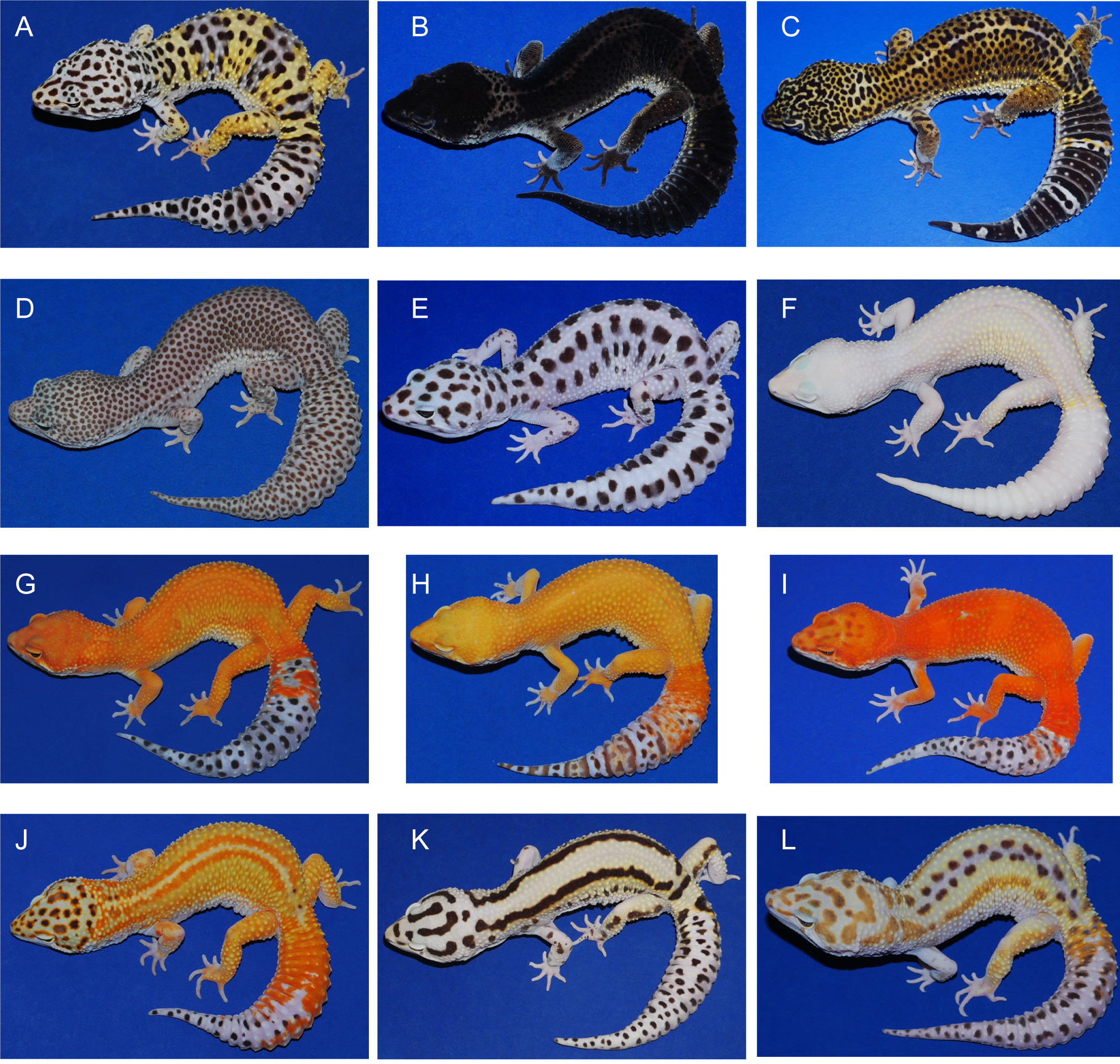

Supplement: S1 Fig — (A) wild type; (B) black night; (C) variant of black night; (D) granite snow; (E) gem snow; (F) white knight; (G) sunburst tangerine; (H-I) variants of sunburst tangerine; (J) red stripes; (K) bold stripes; (L) rainbow. (TIF) [file pgen.1009580.s001.tif]

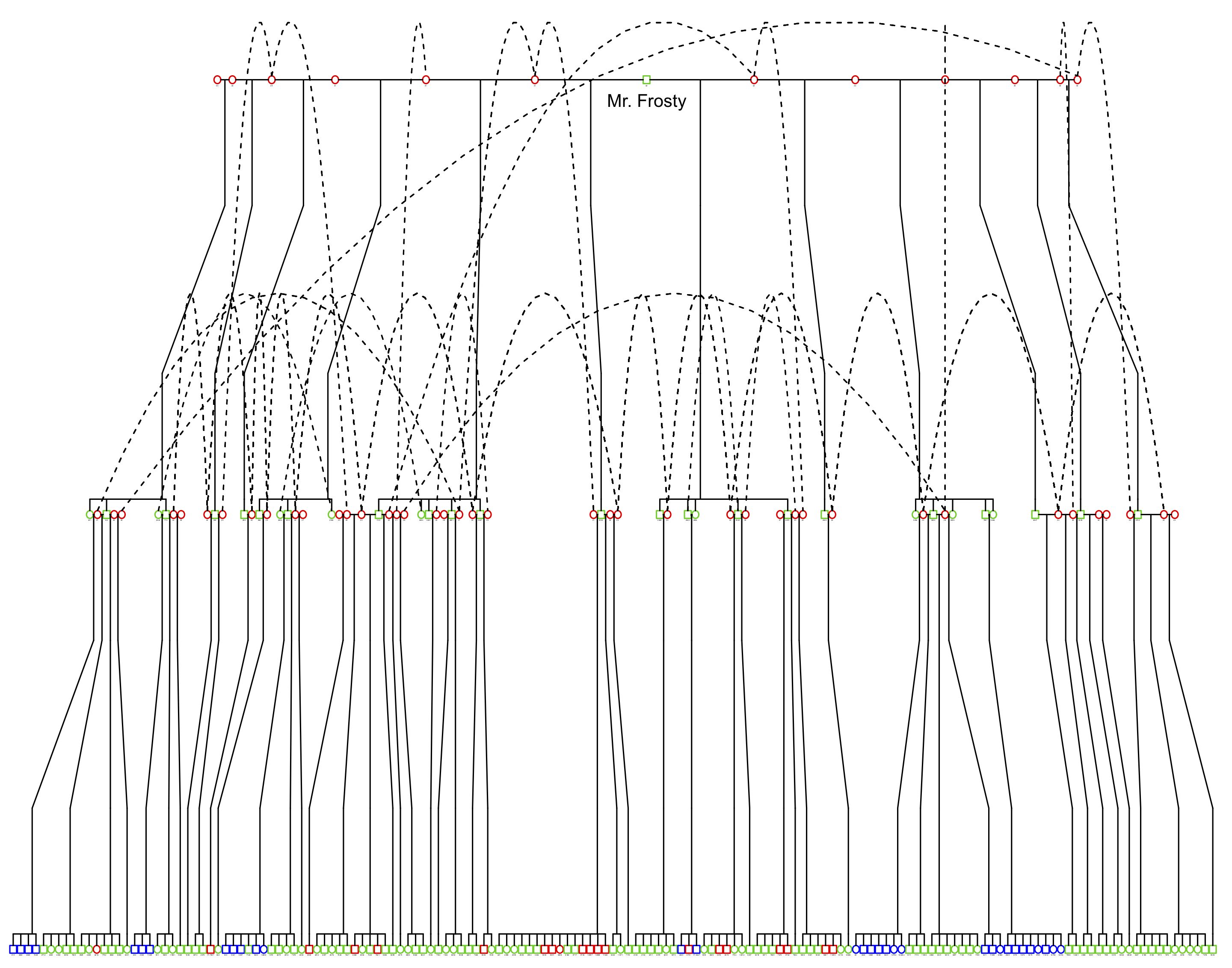

Supplement: S2 Fig — Mr. Frosty, the original carrier of the spontaneous Lemon Frost mutation, was bred to 12 female geckos from different genetic backgrounds. F1s carrying the lf allele were bred among themselves or back to their female parent, producing the second generation of animals heterozygous or homozygous for the lf allele. Blue: lf/lf; green: lf/+; red: +/+. Dashed line: same individual/line. (TIF) [file pgen.1009580.s002.tif]

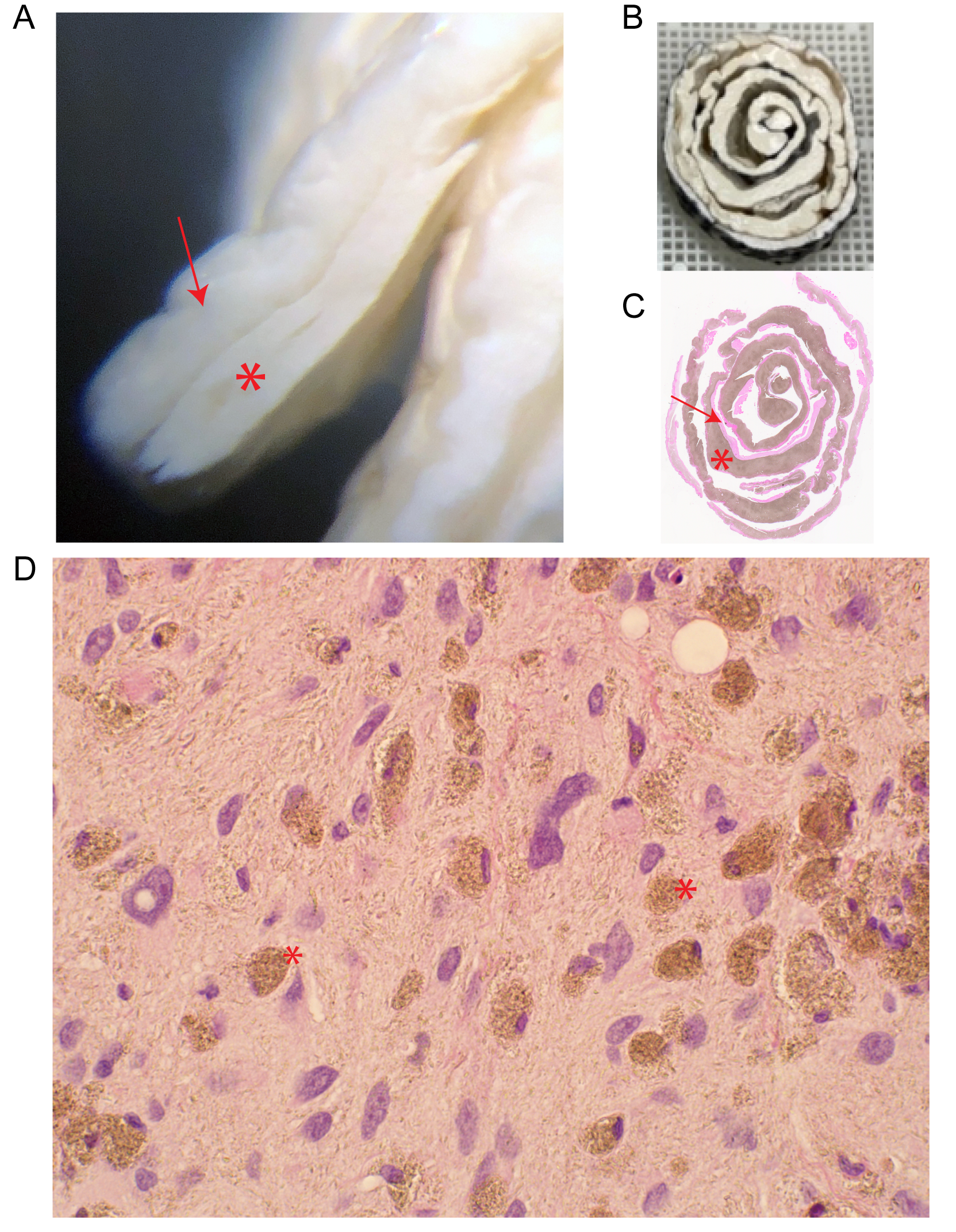

Supplement: S3 Fig — (A) Thick layers of white tumor tissue (star) infiltrating white skin (arrow). (B) Skin biopsies organized and fixed in a paper roll for sectioning. (C) H&E staining of the skin sections. Arrow: skin; star: infiltrated tumor mass. (D) H&E staining of the skin sections showing normal skin cells and neoplastic cells (star). Neoplastic cells have eccentric and condensed nuclei. (TIF) [file pgen.1009580.s003.tif]

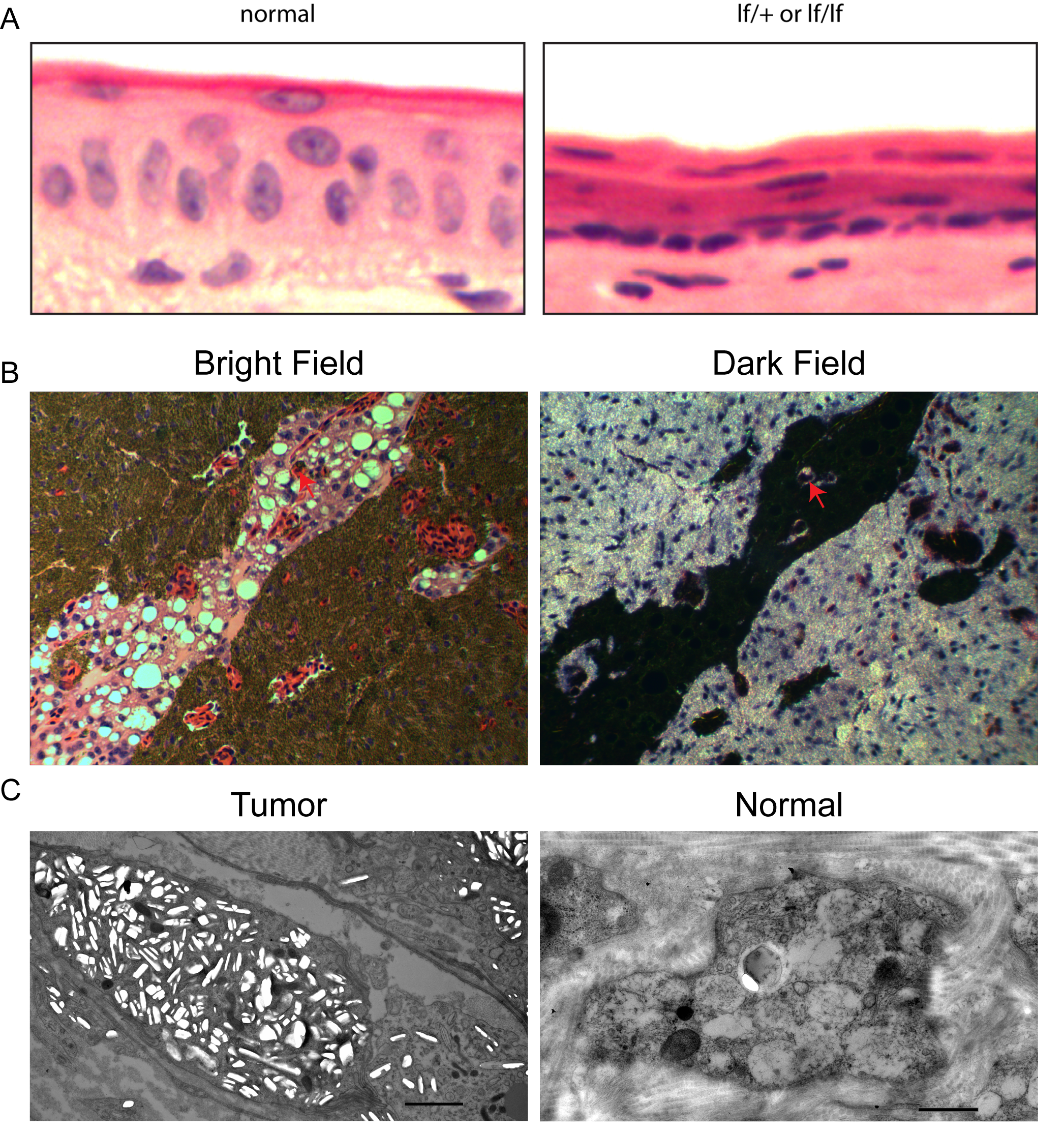

Supplement: S4 Fig — (A) In normal skin, cell nuclei are oval and perpendicular to the skin surface. In Lemon Frost skin, cell nuclei are flat, elongated and parallel to the skin, reminiscent of epithelial-to-mesenchymal transition. (B) Iridophoroma in the liver, stained dark in H&E sections. In dark field imaging, iridophores are bright white. Such iridophores invade blood vessels in the tissue (red arrows). (C) In TEM imaging, white tumor skins in super LF are filled with abundant iridophores with excessive brightly reflective crystals (Tumor). In normal skin, iridophores are much fewer and have less crystals (Normal). (TIF) [file pgen.1009580.s004.tif]

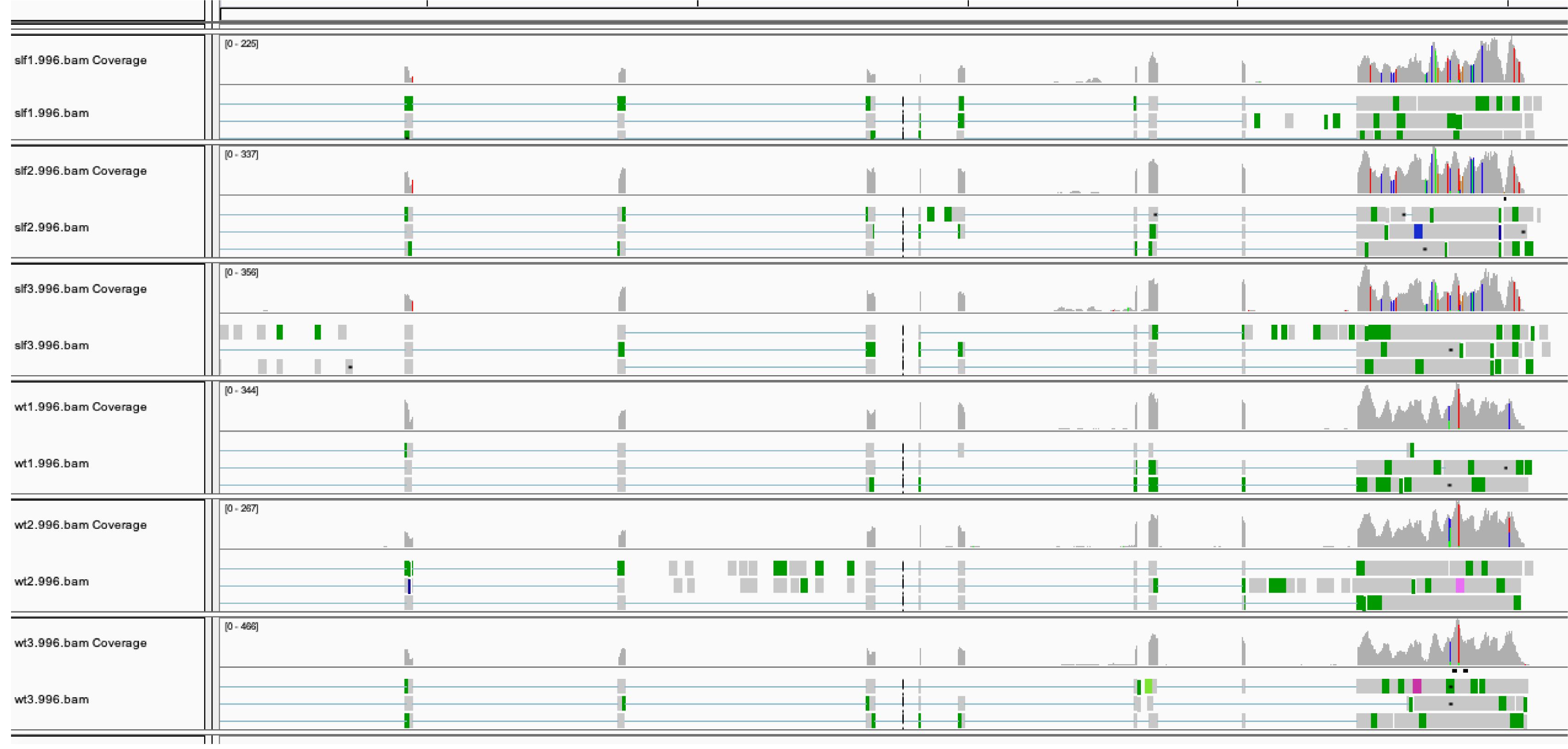

Supplement: S5 Fig — SPINT1 mRNA reads from transcriptome sequencing were aligned to the genome and visualized in IGV. Top 3 rows show samples from homozygous mutants. Bottom 3 rows show samples from wild type geckos. Skin tissue adjacent to the tumors was used in the mutants. Peaks mark SPINT1 exons. The last exon on the right is transcribed together with the 3’UTR. (TIF) [file pgen.1009580.s005.tif]
