# Supplementary material for: Genetics of white color and iridophoroma in “Lemon Frost” leopard geckos
Source: PLoS Genet. 2021 Jun 24;17(6):e1009580. doi: 10.1371/journal.pgen.1009580 (PMC8224956; doi:10.1371/journal.pgen.1009580)
Supplement: Attachment — Submitted filename: reviewer_comments3_final.docx [file pgen.1009580.s010.docx]

Both reviewers found that this work was significant given few mapping studies have been conducted in lizards and they found the approach to be rigorous. Both also are convinced that the SPINT1 gene is a strong candidate gene, and agree that functional validation, which would be important in a model system, is unreasonable to ask in a non-model system. However, given there is no functional validation and little new experimental evidence (beyond a strong statistical association), both reviewers ask that the language about the role of SPINT1 be toned down (e.g. Line 214-215).

Authors: We agree and have toned down the statement as requested. The relevant sentence (lines 247-248 in the revision) now reads, “Our data suggest that an evolutionarily conserved gene, SPINT1, regulates the proliferation of white iridophores in the leopard gecko.”

In addition, Reviewer 2 suggests one additional analysis to examine conservation of the non-coding sequence variants you identified in Lemon Frost to determine if any are likely be functionally important.

Authors: We performed this analysis and added the findings to the results section of the revised manuscript (lines 189-200). Briefly, we found that the leopard gecko intron 1 and 3’UTR are conserved in several other reptile genomes. In human and mouse, introns 1 and 2 and the 3’UTR are regions that contain the most putative enhancers. We were not able to determine whether any of the non-coding sequence variants in Lemon Frost intron 1 and 3’UTR disrupt functionally important sequences, due to the lack of sequence identity between leopard geckos and mammals. We suggest that variants in intron 1 or the 3’UTR may be functionally important.

This seems quite straightforward, and would help round out your results. Finally, Reviewer 1 makes several useful suggestions to improve the structure of the paper, which will likely better highlight and broaden the appeal of your work.

Authors: We restructured our introduction and discussions as suggested.

Lines: 41-45. We expanded the introduction by adding a few sentences in the first paragraph on iridophores, how color in iridophores is created by reflective platelets, and iridophore contribution to organism coloration patterns.

Lines: 60-67. We added a new paragraph on iridophoroma, emphasizing its frequent occurrence in reptiles and our lack of a molecular understanding of the underlying mechanisms. We included the previous description of the Lemon Frost phenotype in this paragraph, which suggested that it can be a good system to study molecular genetics of iridophoroma.

We added the additional analyses to examine conservation of the non-coding sequence variants to the results section (lines 189-200). We also added sentences for a more balanced presentation of both coloration and iridophoroma (lines 108-110,248-250).

**Comments to the Authors:
Please note here if the review is uploaded as an attachment.**

Reviewer #1: Using a combination of techniques, Guo et al. map the genetic basis of the Lemon Frost phenotype, which occurred spontaneously in a Leopard Gecko, to a small genomic region. This region contains only one gene (SPINT1), and, strikingly, this gene makes for an excellent candidate gene, given its known function as a tumor suppressor gene and the high incidence of iridophoromas in individuals carrying the Lemon Frost allele.
I think the authors’ main conclusions that a variant in SPINT1 likely underlies the LF phenotype is supported by their data and, overall, I very much enjoyed reading this manuscript. I congratulate the authors on their work. That being said, I think the manuscript would benefit from a bit of re-structuring (e.g. previous results presented in results and not introduction) and it seems a bit unbalanced in regard to its two central themes of “white coloration” and “iridophoroma” (see below). There are also a few additional points that the authors should/might want to address (e.g. synteny analysis).

Comments:

- I really appreciate that the authors made an effort to keep the introduction short and concise. That being said, I think there is an opportunity for the authors to flesh out the main motivation to investigate the genetic basis of coloration (especially iridophore-based) a bit more. The authors are already doing this to some extent, but, at the moment, the motivation of “There have been few molecular genetic analyses of the regulation of chromatophores in cells other than melanocytes” (ll. 41-42) could maybe be strengthened to draw readers’ attention. Consider adding one or a few sentences to expand on what can be learned from studying coloration and iridophores (i.e. expanding on the points in ll. 42-45 and more generally what is and what is not known). Also, and maybe more importantly, there is no mention of the link between the Lemon Frost phenotype and iridophoromas in the entire introduction (although it is mentioned in the abstract). Given that this is a major part of the results/manuscript, I think the manuscript would greatly benefit from a general paragraph on iridophoromas in the introduction.

Authors: We restructured the introduction as suggested. We added a few sentences (lines: 41-45) in the first paragraph on iridophores, how color in iridophores is created by reflective platelets, and iridophore contribution to organism coloration patterns. We added a new paragraph (lines: 60-67) on iridophoroma, emphasizing its frequent occurrence in reptiles and our lack of a molecular understanding of the underlying mechanisms. We included the previous description of the Lemon Frost phenotype in this paragraph, which suggested that it can be a good system to study molecular genetics of iridophoroma.

- Related to my point above, at the same time, essentially the entire discussion seems to be about iridophoromas and hardly touches upon the genetic basis of white coloration at all. I understand that the two are intricately linked for this phenotype, but I really think that there could be more of a balance of the two themes (coloration and iridophoromas). For example, the Lemon Frost phenotype exhibits a striking banding pattern, but this is hardly (at all) discussed in the entire manuscript.

Authors: We added sentences to the discussion for a more balanced presentation of both coloration and iridophoroma (lines 248-250). We note that the banding pattern is not caused by the Lemon Frost mutation but is associated with the genetic background that Lemon Frost was crossed to. That’s why we also studied a second genetic background (blizzard; Fig. 1D,E). A new sentence was added in the last paragraph of discussion to expand on the discussion of how coloration may be regulated by SPINT1: “Increased production of reflective platelets in Lemon Frost iridophores is a novel finding that warrants further investigation, for instance by manipulating SPINT1 expression.”

- ll. 63-66: “A spontaneous mutation occurred in a female hatchling from a cross between two wildtype leopard geckos. This mutation increased the white color of the leopard gecko, resulting in brightened white and yellow colors. This unique color morph was named Lemon Frost(19)”.
-> I recommend moving this information to the introduction. It seems a bit misplaced in the results section, since the Lemon Frost phenotype apparently first occurred around 2015 and has been described before (reference 19). I think moving these sentences would help readers to understand what is new to this study and what was known before.

Authors: We agree with the reviewer. We have moved this section to the introduction (lines 63-65).

- ll. 81-95: Similar to above, it seems that the authors are at least to some extent reporting previously published results here (references 19 and 24). I think it would be helpful if the authors tried to make it more explicit what exactly was done in this study and how their new data add to previously published results (e.g. do the authors provide new histological details specific to homozygous mutants, more quantitative data, etc.)

Authors: We have moved information on reference 19 to the introduction (lines 63-65). Reference 24 is a general reference on reflective platelets which create color in iridophores. We have added this information and citation to the first paragraph of the introduction as well (lines 40-45). The previous study (reference 19) reported only the occurrence of iridophoroma in 3 Lemon Frost animals with histological diagnosis.

- ll. 91-92: “Microscopy (TEM) showed that the lf allele led to both increased numbers of neoplastic iridophores and increased production of reflective platelets within each iridophore”
-> Does this (increased number of iridophores and increased production of platelets) tell us anything about the mechanism and is it consistent with the function of SPINT1? Unless I missed it, it seems that the increased production of reflective platelets is not mentioned in the discussion at all. Maybe the authors can come back to this result in the discussion.

Authors: Yes, increased number of iridophores and increased production of platelets are the mechanisms of increased white color. We added this sentence to the end of the paragraph to highlight this (lines 108-110): “The increased number of iridophores and increased production of reflective platelets within the iridophores are the likely mechanisms of increased white color in the Lemon Frost morph”

An increased number of iridophores is consistent with the tumor suppressor function of SPINT1. The increased production of platelets is a novel finding. How SPINT1 loss of function may lead to increased number of platelets is unclear. This is now mentioned in the last paragraph of discussion(lines 248-250): “Increased production of reflective platelets in Lemon Frost iridophores is a novel finding that warrants further investigation, for instance by manipulating SPINT1 expression”.

- ll. 127-130: “We found that 17 out of 22 scaffolds that have synteny information (including scaffolds 6052 and 996) correspond to one region on chicken chromosome 5 and human chromosome 15 (Figure 129 3A-C, Supplemental Table).”
-> Do the authors have any explanation for the other five scaffolds? It seems that four of them are in synteny with chr. 7 in chicken. Given the inheritance pattern, I think the main conclusion is probably robust and I highly doubt that there is a second fl locus. It is probably more likely that this due to a chromosomal rearrangement. Nonetheless, given that this block of four scaffolds includes scaffold707, which shows the third highest association signal (Supplementary Table), I think the authors need to mention/discuss this further. It might even be worth it for the authors to expand their synteny analyses to other reference genomes (e.g. Anolis carolinensis or the recently published high-quality Goodes thornscrub tortoise genome).

Authors: We have extended the synteny to the recently improved Anolis (https://www.ebi.ac.uk/ena/browser/view/GCA_000090745.2 ) and tortoise genomes (<https://vgp.github.io/genomeark/Gopherus_evgoodei/>). Three of the four scaffolds (707, 2426, 2893, 4038) with synteny to chicken chr7, along with other scaffolds with synteny to chicken chr5, have synteny to chromosome 1 in Anolis.

We added a sentence in the main text (lines 142-147): “We found that 17 of the 22 scaffolds that have synteny information (including scaffolds 6052 and 996) correspond to one region on chicken chromosome 5, human chromosome 15, and green anole chromosome 1 (Figure 3A-C, Supplemental Table). Three additional scaffolds also have synteny to the green anole chromosome 1. The remaining two scaffolds without synteny to the green anole chromosome 1 were more weakly associated with coloration.”

- ll. 214-215: “We found that an evolutionarily conserved gene, SPINT1, regulates the proliferation of white iridophores in the leopard gecko.”
-> Given that the authors have so far only established association, this statement seems too strong (i.e. it implies functional validation) and should be toned down.

Authors: We agree and have toned down the statement as requested. The relevant sentence (lines 247-249) now reads, “Our data suggest that an evolutionarily conserved gene, SPINT1, regulates the proliferation of white iridophores in the leopard gecko.”

- ll. 244-246: “Lemon Frost and super Lemon Frost phenotypes were determined according to a list of rules, based on increased white color of the body, eye, and belly compared to normal wildtype animals ([http://www.geckosetc.com/lemon_frost_info.html).”](https://urldefense.com/v3/__http:/www.geckosetc.com/lemon_frost_info.html).**B__;4oCd!!F9wkZZsI-LA!T_X1iLAoPhjODhJE3BwYZftRLuh_rpsDxL6CFOLro6DB0bWGt-Kc295OQoxZpUe6ItrQw5Q$)
-> Please note that the link does not work (returns “The page you requested could not be found.“). In any case, I think it would be important if the authors described their phenotyping approach in the manuscript rather than just referring to an external source/website.

Authors: We apologize; the link included a step-by-step guidance to phenotyping with illustrations, as well as a history of Lemon Frost breeding. Unbeknown to us, the breeder took it down. The rules used in phenotyping are now more fully described in the Methods (lines 279-285). The link is now deleted.

- Methods: Please provide version numbers for all software (e.g. GATK, vcftools, etc.)

Authors: Version numbers are now added (lines 297-301).

- l. 262: “All variants were filtered with VCFtools.” and l. 305 “Variants were identified with GATK and filtered with VCFtools.”
-> Filtered how? Please provide details. Is this is described somewhere else, please add “see below” or something alike to help readers find this information.

Authors: More details of variant filtering are now added (lines 301-304). “First, variants had to be bi-allelic and heterozygous in Mr. Frosty. The ratio of the the reads for the two alleles was required to be in the range 0.4-0.6. Second, only high-quality variants were used in homozygosity mapping or statistical mapping (DP>=30, GQ>=30).”

- Supplementary table
-> The supplementary table is missing any sort of annotation. Please add headers inside the table for each sheet, explaining abbreviations, etc.

Authors: Headers are now added.

Reviewer #2: In this study Guo and colleagues investigate the genetic basis of Lemon Frost, a Leopard gecko mutation that results in a white color phenotype and skin tumors. Breeding experiments demonstrate a semi-dominant pattern of inheritance from a single locus. By performing RAD-seq on individuals from a breeding pedigree, Guo et al. localize Lemon Frost to region of the gecko genome that is syntenic to chicken chromosome 5 and human chromosome 15. Whole-genome sequencing of pooled DNAs from wt, heterozygous and homozygous individuals enabled the authors to further localize the mutation to region that contains the SPINT1 gene. However, no nonsynonymous coding mutations were found in SPINT1 and transcriptome analyses of homozygous mutant skin did not reveal changes in SPINT1 expression level or splicing. Nevertheless, functional studies of SPINT1 in zebrafish and the association of SPINT1 deficiency with Skin Cutaneous Melanoma (SKCM) in humans makes SPINT1 an extremely compelling candidate gene for Lemon Frost. The study is well designed and the authors’ statement in the abstract that SPINT1 is a “strong candidate gene” is generally well-supported. As the authors point out, very little molecular genetic research has been performed in reptiles. Therefore, it is exciting this work on a gecko model that may be relevant for understanding the biology of SKCM. Although functional validation of the candidate gene in geckos is currently not practical, I would still like to see some additional evidence that mutation of SPINT1 is responsible for the Lemon Frost phenotype.

Comments
1) Are any of the sequence differences found on the Lemon Frost allele of SPINT1 in well-conserved sequences? Is there evidence that any of the non-coding differences are in regions that are functionally conserved? There are many squamate genomes available (including at least one other species of gecko). This analysis should be relatively straightforward to perform and would not require the generation of additional data.

Authors: We performed this analysis and added the findings to the results section of the revised manuscript (lines 189-200). Briefly, we found that the leopard gecko intron 1 and 3’UTR are conserved in several other reptile genomes. In human and mouse, introns 1 and 2 and the 3’UTR are regions that contain the most putative enhancers. We were not able to determine whether any of the non-coding sequence variants in Lemon Frost intron 1 and 3’UTR disrupt functionally important sequences, due to the lack of sequence identity between leopard geckos and mammals. We suggest that variants in intron 1 or the 3’UTR may be functionally important.

2) Sequencing a more diverse collection of leopard gecko samples might help distinguish which sequence alterations are unique to the Lemon Frost allele. Are there significant barriers to doing this?

We agree that this is an interesting potential future direction, but it is unclear what collection of gecko samples would well capture the sequence diversity, and the genome is large enough that sequencing to the depth needed especially for detection of less-frequent variants would be an expensive endeavor.

3) In the last paragraph of the discussion, the authors’ state “We found that an evolutionarily conserved gene, SPINT1, regulates the proliferation of white iridophores in the leopard gecko.” While this statement is probably true, definitive evidence is not presented in the manuscript - neither protein coding changes nor expression changes in SPINT1 were identified. The authors should tone down this statement.

Authors: We agree and have toned down the statement as requested. The relevant sentence (lines 247-248) now reads, “Our data suggest that an evolutionarily conserved gene, SPINT1, regulates the proliferation of white iridophores in the leopard gecko.”
